# Making Removals Part of Informed Choice: A Mixed-Method Study of Client Experiences With Removal of Long-Acting Reversible Contraceptives in Senegal

**DOI:** 10.9745/GHSP-D-22-00123

**Published:** 2022-10-31

**Authors:** Aurélie Brunie, Fatou Ndiaté Rachel Sarr Aw, Salif Ndiaye, Etienne Dioh, Elena Lebetkin, Megan M. Lydon, Elizabeth Knippler, Sarah Brittingham, Marème Dabo, Marème Mady Dia Ndiaye

**Affiliations:** a FHI 360, Washington, DC, USA.; b IntraHealth International, Dakar, Senegal.; c Centre de Recherche pour le Développement Humain, Dakar, Senegal.; d FHI 360, Durham, NC, USA.; e Department of Surgery, Duke University School of Medicine, Durham, NC, USA; formerly of FHI 360, Durham, NC, USA.; f Sénégal Ministère de la Santé et de l'Action Sociale, Direction de la Sante de la Mère et de l'Enfant, Division Planification Familiale, Dakar, Senegal.

## Abstract

**Background:**

Ensuring access to removal services for implants and intrauterine devices (IUDs) is essential to realize informed choice and voluntary family planning. We document removal desires and experiences among women who received an implant or IUD from the public sector in 3 districts of Senegal.

**Methods:**

We conducted a phone survey of 1,868 implant and IUD users, 598 follow-up surveys with those who had ever asked a provider for a removal, and 24 in-depth interviews (IDIs) with women who had ever wanted an implant removal. We analyzed survey data descriptively and IDI data thematically.

**Results:**

Fifty-eight percent of implant users and 54% of IUD users reported having wanted a removal. Desired pregnancy and contraceptive-induced menstrual changes (CIMCs) were the main reasons for removal desires. Fifty-four percent of implant users and 55% of IUD users who asked a provider for a removal reported challenges accessing services, with over two-thirds noting long lines or wait times. Sixty-three percent of implant users and 73% of IUD users who saw a provider were satisfied with the outcome of their first interaction. Over 90% of participants had not been told about the removal cost at insertion. Almost all participants who had their method removed obtained a complete removal during their first clinical procedure. Around two-thirds of participants who obtained a removal did not take up another method at that time. IDIs confirmed the influence of CIMCs on removal desires and show some partner influence is common in removal decision making. Barriers include lack of available qualified providers and supplies. Provider interactions play an important role in satisfaction with removal services.

**Conclusion:**

Participants’ experiences accessing removal services were generally positive. Areas of potential improvement include client flow, counseling messages at insertion, and when advising clients to keep their method, pricing, and post-removal reinsertion or method switching.

## Abstract (precis)

Governments must plan to ensure that removal services for long-acting reversible contraceptives are accessible and affordable to handle growing demand. Participants seeking method removal in Senegal reported largely satisfactory experiences, with a few areas for potential strengthening.

## Teaser

**Key Findings:**

- Most study participants were satisfied after first interacting with a provider about a removal; however, while the majority of participants who had their method removed were satisfied, most participants who kept their method were dissatisfied.
- Areas of potential improvement to further strengthen access to removal services in Senegal include improving client flow, strengthening counseling messages at insertion and when advising clients to keep their method and on uptake of a new method after removal, as well as lowering pricing.

**Key Implications:**

- Similar strategies can be deployed to strengthen removal services for both implants and intrauterine devices.
- Program managers should reinforce counseling on side effects and on post-removal method switching and reinsertion.
- Policy makers should develop guidance to harmonize service fees across facilities and review procurement and funding mechanisms for supplies to support continued service availability and ensure costs are not passed on to clients.

Résumé en français à la fin de l'article.

## INTRODUCTION

Trends in the contraceptive method mix in sub-Saharan Africa reveal a progression among hormonal contraceptives of pills to injectables to implants as the leading method in the mix.^1^ Implant use has grown tremendously, both in prevalence and share of the method mix. Between 2014 and 2020, implant procurement more than doubled in sub-Saharan Africa.^2^ Currently, implants have surpassed injectables to become the leading method among married women in Benin, Burkina Faso, Guinea-Bissau, Mali, Rwanda, and Senegal.^3^ Moreover, increased implant use has driven gains in modern contraceptive prevalence in 11 countries.^4^

Removal of long-acting reversible contraceptives (LARCs), including implants and intrauterine devices (IUDs), is essential to fulfill informed choice, women’s reproductive autonomy, and a rights-based approach to care by allowing users to decide not only when to start but also when to discontinue their chosen method.^5^^,^^6^ Growth in implant use will unavoidably accelerate the need for removal, with a lag time reflecting up to the 3-to-5-year lifespan of current products or less if users choose to remove their method earlier.^7^ Using procurement data and assuming implants would be used for their couple-years of protection unit (2.5 years for Implanon and 3.8 years for Jadelle), Christofield and Lacoste estimated that the number of removals would more than double between 2015 and 2018.^7^^,^^8^ Demographic and Health Survey data from several countries show that a sizable number of women discontinue implants in their first year of use,^9^ potentially leading to faster growth in demand for removals.

Learnings from the scale-up of the 6-rod Norplant system in the 1990s highlight shortcomings in access to and quality of removal services, which, in turn, had negative repercussions for reputation and uptake of the method.^10^^–^^12^ In contrast to the growth in implant use, IUD use has declined overall in sub-Saharan Africa.^1^ Despite this decline, ensuring accessible, affordable, and good quality removal services remains important to uphold voluntary family planning. Few studies have examined access to removals in the context of second-generation products like Jadelle and Implanon NXT,^13^ and evidence on IUD removals in low- and middle-income country settings is even scanter. Recent studies of user experiences with LARC removals in Ethiopia, Ghana, and Kenya reported some challenges, including provider barriers, cost, difficult removals, and transportation issues.^14^^–^^16^ This study conducted in Senegal seeks to extend this growing body of evidence to Francophone West Africa, spanning both implant and IUD removals.

Modern contraceptive prevalence among married women increased from 12% to 26% between 2011 and 2019 in Senegal.^17^^,^^18^ During the same period, implant use grew from 9% to 38% of the modern contraceptive method mix and use of the copper IUD, the only available IUD, minimally increased from 5% to 7%.^17^^,^^18^ Overall, 96% of implants and 90% of IUDs are sourced through the public sector.^17^ In the public health system, the costs to clients include a regulated amount for the product, a service fee fixed by facilities at insertion, and a service fee at removal. Available estimates indicate that 11% of implant users discontinue their method within the first year of use.^17^ The objectives of our study were to document LARC removal desires, describe LARC removal outcomes, and document barriers to removals from the perspective of women procuring their method through the public sector along their journey to accessing removal services.

From 2011 to 2019 in Senegal, implant use increased from 9% to 38% of the modern contraceptive method mix.

## METHODS

### Study Design and Data Collection

We conducted a cross-sectional, mixed-method study to retrospectively examine LARC users’ experiences in 3 districts of Senegal purposively selected to introduce geographical and cultural variation (Dakar Centre, Kolda, and Saint-Louis). Dakar Centre is a primarily urban district that includes the capital city Dakar. Located north of Dakar near the mouth of the Senegal river, Saint-Louis has an important tourist industry and is a commercial and industrial center for sugar production. Kolda is part of one of the country’s most rural regions in the South. Despite this, a 2017 assessment found that only 24% of households in Saint-Louis region live within 1 km of a health facility compared to 50% in Kolda region; in most cases, the closest facility is a health post (87% of cases in Kolda and 62% in Saint-Louis).^19^

This study was part of a larger project that also examined provider experiences with removal services; provider results are presented elsewhere.^20^ Eligible participants were adults or emancipated minors who had an implant or IUD inserted at a public health facility between July 2016 and June 2018 and had phone information available in their clinic records. Providers identified all eligible participants from clinic registers, called them to inform them of the study, and provided the research team with the information of those agreeing to be contacted. Based on available information and local expert knowledge, we assumed that 87% of women in Dakar, 69% in Saint-Louis, and 43% in Kolda would have phones; that clinic records would have phone information for 35% of these women; that providers would reach 50% of women with phone information available; and that 90% of the women reached would agree to participate in the study. Using service statistics on the numbers of implant and IUD insertions, we anticipated being able to survey 1,706 implant acceptors and 566 IUD acceptors.

Data collection involved a phone-based population survey of participants willing to be contacted, followed by an in-person survey or in-depth interview (IDI) with the subset of participants indicating during the phone survey that they had ever asked a provider to remove their method. For IDIs, we also included participants who had ever wanted a removal but never asked a provider for one. The follow-up survey was planned in person to improve data quality based on the advice of local investigators. Due to the coronavirus disease (COVID-19) pandemic restrictions, we conducted follow-up interviews by phone in Saint-Louis. We selected implant users only for IDIs because this method is more widely used. We used responses to the phone survey to purposively select IDI participants representing a range of removal outcomes and invited all other eligible participants to participate in the follow-up survey. The follow-up survey was to be conducted within 1–4 weeks of the phone survey.

The phone survey examined sociodemographic characteristics, counseling received at insertion, whether respondents had ever wanted and/or tried to get their LARC removed, the number of attempts made, and, for participants who never asked a provider for a removal, their experiences with their method. The follow-up survey and IDIs covered experiences with the method and a review of each removal attempt made to date, spanning the journey from decision making to removal procedure, as applicable.

Interviews were conducted in French, Poular, Socé, or Wolof. Trained research assistants used tablets to conduct the phone survey between December 3, 2019 and March 31, 2020. The follow-up survey was conducted between January 24, 2020 and March 19, 2020 in Dakar and Kolda districts and May 11, 2020 and June 1, 2020 in Saint-Louis district. Separate research assistants conducted, audio-recorded, and transcribed IDIs into French. Participants gave their oral consent for phone interviews and written consent for in-person interviews. We compensated participants 1000 West African CFA francs (CFA) (US$1.80) as mobile money for the phone survey and CFA5000 (US$8.93) for follow-up interviews.

### Ethical Approval

The Comité National d’Éthique pour la Recherche en Santé in Senegal and FHI 360’s Protection of Human Subjects Committee in the United States approved the study.

### Analysis Methods

We analyzed data descriptively by LARC using Stata (Version 16.1) and SAS Enterprise Guide (Version 8.2). We determined implant type based on responses to questions on the number of rods and the duration of protection stated by the provider at insertion.

In reporting removal outcomes, we defined a removal attempt as discussing removal with a provider. A situation whereby a participant traveled to the facility but was unable to see a provider was not counted as a removal attempt. Conversely, not all removal attempts may have involved a removal procedure as participants may have been counseled to keep their method. Because participants who still had their method may seek to remove their method again at a later point in time, the primary focus of our analyses on experiences with removals was on the first removal attempt. Our definition of a successful attempt is based on participants’ stated satisfaction with the outcome of the interaction with the provider, regardless of whether they kept the method or had it removed. Satisfaction was measured by asking whether participants were happy with keeping/removing their method.

Because participants who still had their method may seek to remove their method again at a later point in time, the primary focus of our analyses on experiences with removals was on the first removal attempt.

We conducted an exploratory multivariable logistic regression analysis to examine factors associated with asking a provider for a removal. This analysis was conducted among implant users only because implants are the most popular of the 2 methods. We included 18 factors related to sociodemographic characteristics, prior method use, partner knowledge of method use at insertion, insertion cost, information received at insertion in terms of counseling and method choice, and experiences with side effects and contraceptive-induced menstrual changes (CIMCs). We created an indicator variable for informed choice with a value of 1 for participants who said that, at insertion, they were informed about other methods and informed about side effects and told what to do if they experienced side effects (method information index) and 0 otherwise. We checked for multicollinearity using variance inflation factors and did not find levels of concern. We used adjusted odds ratios (AOR) with accompanying 95% confidence intervals (CIs) and assessed significance at the 5% level to examine associations based on the logistic models.

Two analysts coded IDI transcripts in NVivo 12 using a thematic codebook and conducting periodic verification of intercoder agreement on approximately 10% of transcripts. We then prepared detailed memos summarizing the dimensions of each main code, as well as matrices to observe patterns in themes by removal outcome and district.

## RESULTS

### Quantitative Results

Providers identified 4,014 eligible women from clinic records, of whom 2,407 agreed to being contacted by the research team. Of these, 1,868 (78%) completed the phone survey. Altogether, 799 women who had ever asked a provider to remove their method were eligible for a follow-up interview; 598 of them (75%) completed the follow-up survey (Figure).

**FIGURE:**
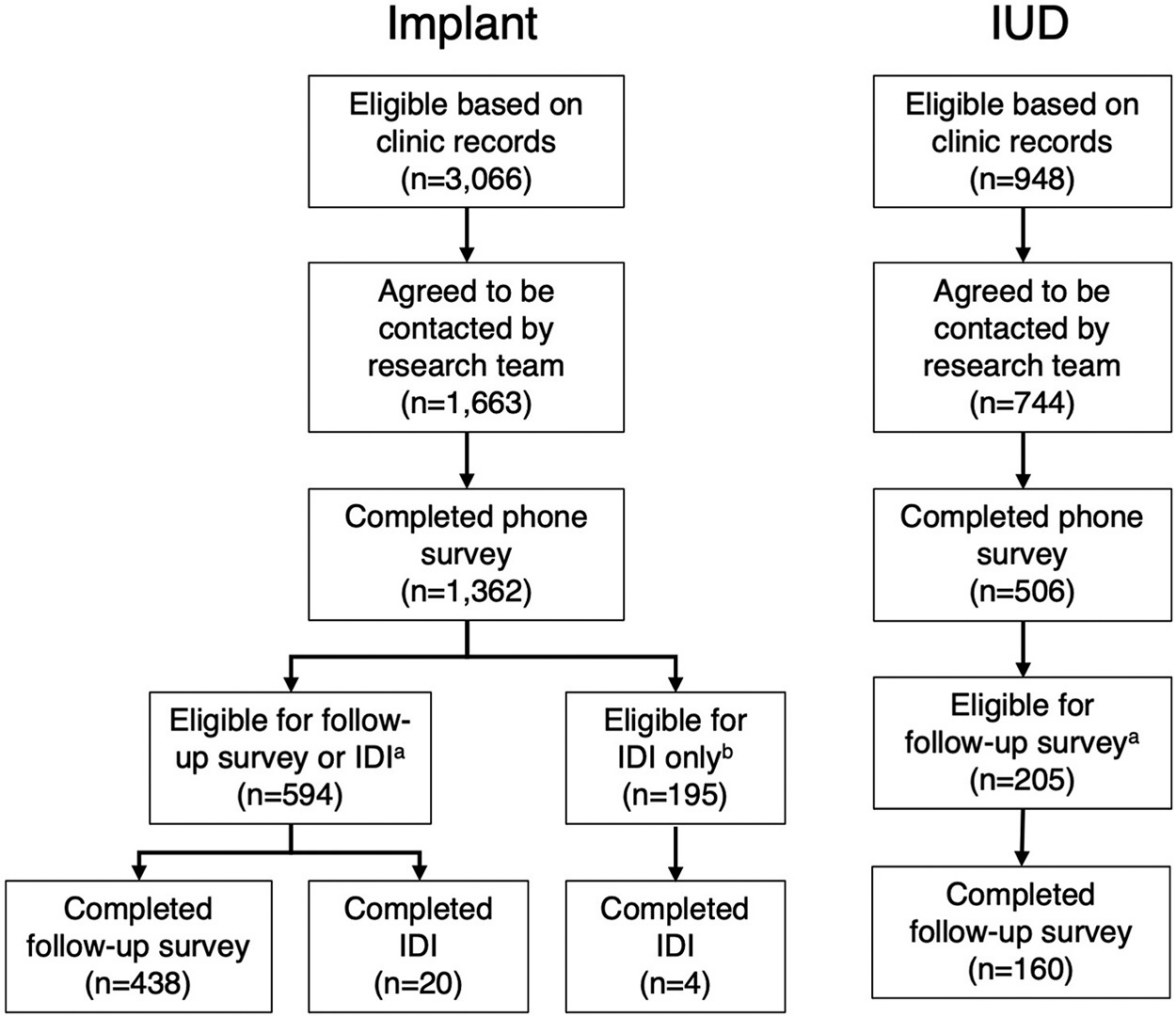
Flow Chart for Implant and IUD Users’ Removal Experiences in 3 Districts in Senegal Abbreviations: IDI, in-depth interview; IUD, intrauterine device. ^a^ Ever asked a provider for a removal. ^b^ Ever wanted a removal but never asked a provider for one.

The mean age was 31 years for implant users and 36 years for IUD users, and the mean parity was 3 (Table 1). More than 88% of participants were married and more than 93% were Muslim, and 72% of implant users and 86% of IUD users were in the upper wealth quintile. Seventy-six percent of implant users and 90% of IUD users had prior experience with modern contraception, and 23% and 20%, respectively, had previously used the same LARC as their study method. On average, implant users had received their method 29 months before the survey and IUD users 30 months. Jadelle was the most common implant type. Notably, 21% of participants gave inconsistent or incomplete information on the number of rods and the duration of protection of their implant, thus leaving us unable to determine their implant type. The mean number of days between the 2 surveys was 51 days for implant users and 49 days for IUD users. To examine potential biases due to attrition between the 2 surveys, we compared the characteristics of eligible participants who completed the follow-up survey to those of eligible participants who did not complete it. The characteristics were similar between the 2 groups with no more than a 5% variation in characteristics noted.

**TABLE 1.** Participant Characteristics and Counseling Received at Implant and IUD Insertion in 3 Districts of Senegal^a^

	**Implant**			**IUD**		
	**Completed Phone Survey** **(n=1,362)**	**Eligible for In-person Survey** **(n=594)**	**Completed In-person Survey** **(n=438)**	**Completed Phone Survey** **(n=506)**	**Eligible for In-person Survey** **(n=205)**	**Completed In-person Survey** **(n=160)**
Age, years, mean (SD)	31.2 (6.8)	29.8 (6.2)	30.0 (6.4)	36.2 (7.3)	35.6 (8.0)	36.2 (7.7)
Marital status, %						
Single	3.7	2.0	1.6	2.0	1.5	1.3
Married/cohabitating	88.6	91.1	92.7	92.7	90.8	90.6
Divorced/widowed	7.8	6.9	5.7	5.3	7.8	8.1
Parity, mean (SD)	2.6 (1.7)	2.3 (1.5)	2.4 (1.6)	3.3 (1.7)	3.2 (1.7)	3.3 (1.7)
Highest education, %						
None	21.7	19.4	19.9	12.5	12.2	10.6
Primary	30.3	29.0	31.3	32.1	33.7	38.8
Middle	16.9	18.7	15.9	17.4	14.7	16.2
Secondary school	16.7	18.0	18.1	15.3	17.6	16.9
Higher than secondary school	14.5	15.0	14.8	22.7	22.0	17.5
Religion, %						
Muslim	95.1	95.6	96.4	93.7	94.2	93.1
Christian	4.9	4.4	3.7	6.3	5.9	6.9
Wealth quintiles^b^, %						
Lowest	4.5	3.5	3.6	2.1	1.7	1.4
Second	3.6	4.3	4.4	1.2	0.6	0.7
Middle	4.4	4.3	4.1	2.1	1.1	0.7
Fourth	15.2	13.8	12.7	8.5	8.6	10.8
Highest	72.3	74.2	75.1	86.2	88.0	86.3
Months since method inserted, mean (SD)	29.4 (6.4)	30.5 (6.4)	30.7 (6.4)	29.5 (6.2)	29.8 (5.6)	29.5 (5.6)
Implant type, %						
Jadelle	61.2	56.7	57.6	N/A	N/A	N/A
Implanon	17.6	19.2	18.7	N/A	N/A	N/A
Unknown	21.3	24.1	23.7	N/A	N/A	N/A
Contraceptive use history, %						
Previous use of current method	22.5	18.9	19.4	19.6	18.5	20.0
Previous use of any modern method	75.9	76.4	77.6	90.1	93.2	94.4
Partner knowledge of current method at time of insertion	86.3	86.9	87.0	82.4	83.4	84.4
Told at insertion that removal can be obtained any time	92.9	94.1	93.6	96.2	97.1	97.5
Told at insertion where removal can be obtained						
Insertion place only, %	55.5	55.1	56.2	55.6	57.1	60.0
Place other than insertion place	0.7	0.8	1.1	0.4	1.0	1.3
Insertion place and another place	27.0	28.3	25.3	24.8	25.9	21.9
Not told about any place	15.4	14.7	16.7	17.8	14.6	15.0
Not sure	1.4	1.2	0.7	1.4	1.5	1.9

Abbreviations: IUD, intrauterine device; N/A, not applicable; SD, standard deviation.

^a^ Data are from phone survey participants. Nonresponses varied across items; small amounts of data are missing.

^b^ Relative wealth was measured using the EquityTool (https://www.equitytool.org/). The national version of the EquityTool compares participants to the national population.

#### Counseling and Experiences Using LARCs

More than 92% of LARC users recalled being told at insertion by the provider that they could remove their method at any time (Table 1). Fifty-five percent or more of participants only recalled being told about the insertion place as a location where they could get a removal.

More participants reported experiencing CIMCs than other side effects (Table 2). Among participants reporting CIMCs, the most commonly reported CIMCs were bleeding disturbances (i.e., changes in frequency, irregular bleeding, spotting) for implants (61%) and heavier bleeding during period for the IUD (55%). Sixty-two percent of implant users and 58% of IUD users with CIMCs said they were concerned it would affect their health. Sixty-three percent of implant users and 65% of IUD users with CIMCs said it had no impact on their daily lives, but 36% and 34%, respectively, reported a negative impact.

**TABLE 2.** Participant Experiences Using Implants and IUDs in 3 Districts of Senegal^a^

	**Implant, %** **(n=1,202)^b^**	**IUD, %** **(n=450)^b^**
Participants reporting CIMCs	77.4	53.6
Type of CIMCs reported^c^^,d^		
Bleed more during period	27.1	55.2
Bleed less during period	12.0	9.1
Period lasts longer	35.6	44.8
Bleeding disturbances^e^	60.8	46.9
Stopped having period	33.1	11.2
Concern with CIMCs^d^		
Very concerned	29.4	27.5
Somewhat concerned	32.5	30.4
Not at all concerned	38.2	42.1
Impact of CIMCs on daily life^d^		
Positive	1.5	0.8
Negative	35.5	33.9
No impact	63.0	65.3
Participants reporting weight gain	37.8	20.1
Participants reporting side effects other than weight gain and CIMCs	37.5	44.2
Type of side effect reported^a^^,f^		
Headaches	31.1	11.1
Weight loss	25.3	6.6
Abdominal pain	29.3	42.9
Dizziness	22.9	9.1
Vaginal infections	N/A	24.8
Pelvic discomfort/pain	N/A	14.7
Impact of side effects on daily life^f^		
Greatly impacted	19.6	18.2
Impacted a little	33.4	30.2
No impact	47.0	51.6

Abbreviations: CIMCs, contraceptive-induced menstrual changes; IUD, intrauterine device; N/A, not applicable.

^a^ Data are from phone and in-person survey participants. Nonresponses varied across items; small amounts of data are missing.

^b^ Due to the design of the questionnaires, this information is not available for participants who said in the phone survey that they asked a provider for a removal but who did not complete the in-person interview.

^c^ Multiple responses possible, spontaneous mention; responses with values of ≥10% reported.

^d^ Among participants who reported CIMCs.

^e^ Bleeding disturbances include irregular bleeding, spotting, and having a period more often.

^f^ Among participants reporting side effects other than weight gain and CIMCs.

#### Removal Desires

Overall, 58% of implant users and 54% of IUD users reported having wanted a removal (Table 3). This included 44% and 41% who had asked a provider for a removal, respectively. Regardless of whether participants had attempted to get a removal, the most common reasons for wanting a removal were desired pregnancy and CIMCs across both methods. Participants who wanted a removal but had not asked a provider for one cited lack of time as the main reason. Among those who had interacted with a provider, 96% of implant users and 89% of IUD users went to the clinic with the intention to remove their method. Of these, 67%–75% reported making the decision to see a provider on their own and 19%–27% said they were influenced by their partner.

**TABLE 3.** Participant’s Reported Desire to Remove Implant or IUD in 3 Districts of Senegal^a^

	**Implant, %** **(n=1,362)**	**IUD, %** **(n=506)**
Reported desire to remove		
Never wanted a removal	42.1	46.0
Wanted a removal but have not asked a provider	14.2	13.4
Asked provider for removal	43.6	40.5
Reasons for wanting to stop using method^b^^,^^c^		
Desired pregnancy	25.8	36.8
Bleeding disturbances^d^	20.6	10.3
Bleed more during period or period longer	11.3	7.4
Reasons for not asking provider^e^^,^^c^		
Busy/no time	44.0	33.8
Changed mind/decided to keep	14.1	16.2
Method came out on own	0.0	13.3
Reasons for wanting to stop using method^b^^,^^f^		
Desired pregnancy	29.9	27.6
Bleeding disturbances^c^	23.4	12.8
Bleed more during period or period longer	15.4	16.7
Partner disapproved	12.7	7.7
Weight loss	12.3	3.2
Weight gain	11.6	1.3
Timing of removal decision^f^		
Decided before coming to facility	95.6	89.0
Decided at facility visit	4.4	11.0
Social influence reported for removal desire^f^		
Self	66.6	75.4
Husband/partner	27.1	18.8
Other^g^	6.3	5.8

Abbreviation: IUD, intrauterine device.

^a^ Data are from phone and in-person survey participants. Nonresponses varied across items; small amounts of data are missing.

^b^ Multiple responses possible, spontaneous mention; responses with values of ≥10% reported.

^c^ Among participants who wanted a removal but have not asked a provider.

^d^ Bleeding disturbances include irregular bleeding, spotting, and having period more often.

^e^ Responses do not total 100% as only responses with at least 10% of participants responding are listed.

^f^ Among participants who have asked a provider for a removal.

^g^ Includes other relative, friend, colleague, community health worker, and other unspecified.

The most common reasons for wanting a removal were desired pregnancy and CIMCs across both methods.

In the multivariable model on factors associated with asking for a removal among implant users, participants who made an informed choice (AOR=1.42; 95% CI=1.03, 1.96), participants who experienced amenorrhea (AOR=1.61; 95% CI=1.12, 2.32), and participants who experienced other non-bleeding side effects besides weight gain (AOR=2.61; 95% CI=1.89, 3.60) had higher odds of asking a provider for a removal, while participants who experienced shorter or reduced bleeding (AOR=0.55; 95% CI=0.33, 0.91) had lower odds of asking for one (Table 4).

**TABLE 4.** Association of Factors With Participants Asking a Provider to Remove Implant in 3 Districts of Senegal^a^

	**AOR (95% CI)** **(n=820** ^b^ **)**
Age group, years	
18–24	Reference
25–34	0.72 (0.45, 1.14)
35–49+	0.65 (0.36, 1.17)
Education level	
None	Reference
Primary/middle	1.04 (0.67, 1.61)
Secondary or higher	0.74 (0.44, 1.24)
Religion	
Christian	Reference
Muslim	1.51 (0.69, 3.30)
Parity	
0	Reference
1–2	0.56 (0.19, 1.66)
3–4	0.41 (0.13, 1.28)
5+	0.34 (0.10, 1.19)
Fertility intentions	
Do not want more children/do not want to have children/unsure	Reference
Want more children/want to have children	1.24 (0.76, 2.04)
Wealth (urban score)	
Low (quintile 1–3)	Reference
Middle (quintile 4)	0.89 (0.57, 1.40)
High (quintile 5)	1.03 (0.68, 1.56)
Previous use of current method	0.74 (0.50, 1.11)
Partner knowledge of current method at time of insertion	1.18 (0.74, 1.89)
Informed choice	1.42 (1.03, 1.96)^c^
Received method wanted	0.81 (0.45, 1.44)
Insertion was free	0.76 (0.54, 1.07)
Informed method could be removed at any time	1.00 (0.55, 1.81)
Experienced amenorrhea	1.61 (1.12, 2.32)^c^
Experienced bleeding disturbances	1.13 (0.81, 1.56)
Experienced shorter or reduced bleeding	0.55 (0.33, 0.91)^c^
Experienced longer or heavier bleeding	1.23 (0.87, 1.75)
Experienced weight gain	0.94 (0.68, 1.30)
Experienced other side effects^d^	2.61 (1.89, 3.60)^c^

Abbreviations: AOR, adjusted odds ratio; CI, confidence interval.

^a^ Data are from phone and in-person survey participants. Nonresponses varied across items; small amounts of data are missing.

^b^ Sample size of implant users (n=1,362) is reduced to n=820 in the multivariable regression model due to missing data.

^c^ Statistically significant (*P*≤.05).

^d^ Other side effects include any mentioned non-bleeding side effects other than weight gain.

#### Access to Removal Services

Eighty-one percent of LARC users who attempted removal returned to the place where they received their method for their first removal attempt (Table 5). Fifty-three percent of implant users and 55% of IUD users who attempted removal reported experiencing challenges accessing removal services. Among those reporting challenges, more than two-thirds experienced long lines or wait times. Twenty percent or more had difficulty getting away from their home or finding money.

**TABLE 5.** Experience Seeking Removals Among Participants Who Asked a Provider for an Implant or IUD Removal in 3 Districts of Senegal^a^

	**Implant** **(n=438)**	**IUD** **(n=160)**
Location of first removal attempt, %		
Same place as insertion	81.5	80.7
Different place	18.5	19.4
Reported facing challenges accessing facility or at facility	53.5	55.1
Challenges faced,^b^^,^^c^ %		
Long line/long wait	67.1	75.6
Difficulty getting away from house	29.4	31.4
Difficulty finding money to pay for transport and services	23.8	19.8
Provider was not available	17.8	10.5
Difficulty finding transport	10.8	4.7
Outcome of first removal attempt, %		
Method removed, reported satisfied to remove	53.3	63.6
Method removed, reported would have preferred to keep	4.0	3.9
Still has method, reported satisfied to keep	9.9	9.7
Still has method, reported would have preferred to remove	32.2	22.7
Partial/failed removal	0.7	0.0
Reasons provider did not remove at first interaction,^b^^,^^d^ %		
Provider counseled to keep method	33.9	50.0
Qualified provider not available	30.6	22.0
Equipment/supplies not available for removal	12.0	2.0
Consultation period over/client arrived late to clinic	11.5	2.0
Provider refused to remove	6.6	14.0
Outcome of most recent removal attempt, %		
Method removed, reported satisfied to remove	81.0	82.1
Method removed, reported would have preferred to keep	6.9	6.4
Still has method, reported satisfied to keep	5.9	6.4
Still has method, reported would have preferred to remove	5.7	5.1
Partial/failed removal	0.5	0.0
Number of attempts until complete removal, mean (SD)	1.4 (0.7)	1.3 (0.5)

Abbreviations: IUD, intrauterine device; SD, standard deviation.

^a^ Data are from in-person survey participants. Nonresponses varied across items; small amounts of data are missing.

^b^ Multiple responses possible, spontaneous mention; responses with values of ≥10% reported.

^c^ Among participants who reported facing a challenge accessing facility or at facility.

^d^ Among participants who reported still having method after first removal attempt.

#### Interaction With Providers

Focusing on the first attempt, 63% of interactions with a provider for implant users and 73% for IUD users resulted in an outcome deemed satisfactory by participants (Table 5). Most participants who had their method removed during this interaction were satisfied (53% versus 4% of all participants who had attempted removal), while most participants who kept their method were dissatisfied (32% versus 10% of all participants who had attempted removal). Among participants who did not obtain a removal, 34% of implant users and 50% of IUD users were counseled by the provider to keep their method, while 31% and 22%, respectively, reported that a qualified provider was not available. At the time of the survey, 87%–88% of participants who had asked for a removal reported a satisfactory outcome. Of those who had asked for a removal, 88% of both implant and IUD users had their method removed, with an average of 1.4 attempts until removal for implant users and 1.3 for IUD users.

On the first attempt, 63% of interactions with a provider for implant users and 73% for IUD users resulted in an outcome deemed satisfactory.

#### Removal Procedures and Post-Removal Contraceptive Use

Almost all participants who had their method removed obtained a complete removal during their first clinical removal procedure (Table 6). Fifty-eight percent of participants who had their implant removed and 36% of those who had their IUD removed reported some complications, primarily temporary pain although 22% of implant users reported pain lasting several days. Additionally, 18% of implant users were told by the provider during the removal procedure that there were difficulties, with the main reason given being that the implant was non-palpable (40 of 67 participants).

**TABLE 6.** Implant and IUD Removal Procedures and Post-Removal Contraceptive Use Among Participants Who Had Their Method Removed at the Time of the Survey in 3 Districts of Senegal^a^

	**Implant** **(n=379)**	**IUD** **(n=136)**
Had method removed during first clinical procedure, %	99.7	100.0
Reported complication at removal, %	58.1	36.0
Complications reported,^b^^,c^ %		
Temporary pain at time of removal	63.6	85.7
Pain/discomfort that lasted throughout the day	33.6	22.5
Pain/discomfort that lasted a few days	21.8	8.2
Told by provider there were difficulties during removal, %	17.7	4.4
Reported duration of removal procedure, mean (range), minutes	14.5 (0–120)	11.3 (0–75)
Reported time spent at facility for removal, mean (range), minutes	81.0 (1–420)	77.7 (0–360)
Reported cost of removal, mean (range), CFA [US$]	1891 (0–20000) [3.21 (0–33.96)]	1327 (0–17000) [2.25 (0–28.86)]
Reported cost of insertion, mean (range), CFA [US$]	1491 (0–9000) [2.53 (0–15.28)]	1118 (0–12000) [1.90 (0–20.37)]
Actual cost for removal compared to removal cost told at time of insertion, %		
More expensive	1.1	0.0
Same price	3.3	2.3
Less expensive	1.4	0.0
Was not told price at insertion	93.3	96.2
Reported ease of overall removal experience, %		
Very easy	36.9	58.8
Somewhat easy	38.0	28.7
Somewhat difficult	17.9	10.3
Very difficult	7.1	2.2
Did not obtain another contraceptive method after removal, %	66.2	64.7
Reasons reported,^b^^,^^d^ %		
Afraid of side effects	19.1	18.2
Partner disapproved	10.4	8.0
Any reason other than desired pregnancy, sexual inactivity, or infecundity	21.9	17.7

Abbreviations: CFA, West African CFA franc; IUD, intrauterine device.

^a^Data are from in-person survey participants except for cost of insertion which is from the phone survey. Nonresponses varied across items; small amounts of data are missing.

^b^Multiple responses possible, spontaneous mention; responses with values of ≥10% reported.

^c^Among participants who reported complications at removal.

^d^Among participants who did not obtain another contraceptive method after removal.

The average reported duration of the removal procedure was 15 minutes for implants and 11 minutes for IUDs, compared to a total time spent at the facility of 81 and 78 minutes, respectively. The average cost participants reported paying for a removal was CFA1891 (US$3.21) for implants and CFA1327 (US$2.25) for IUDs, compared to CFA1491 (US$2.53) and CFA1118 (US$1.90) reported by the same participants for insertions. Between 93% and 96% of participants who had their method removed said they had not been told what the cost of a removal would be at the time of insertion. Altogether, 75% of implant users and 88% of IUD users who had their method removed rated their overall experience (from the time they decided to remove their method until removal) as easy, but 7% and 2%, respectively, rated it as very difficult.

Among participants who obtained a removal, 66% of implant users and 65% of IUD users did not take up another contraceptive method during the same visit. Reasons these women did not take up another method included desiring pregnancy (22%) and no sexual activity (18%). Other commonly mentioned reasons included infecundity with fear of side effects (19% of implant users and 18% of IUD users) and partner disapproval (10% of implant users and 8% of IUD users).

Among participants who obtained a removal, around two-thirds of implant and IUD users did not take up another contraceptive method during the same visit.

### Qualitative Results

We completed 24 IDIs with implant users, including 6 with participants who had their implant removed after 1–2 interactions with a provider and declared themselves satisfied, 6 with participants who still had their implant after 1–2 interactions with a provider and were satisfied, 8 with participants who had more than 2 interactions with a provider (regardless of the outcome), and 4 with participants who said they had wanted to remove their implant but had never interacted with a provider about a removal.

#### Removal Desires

Many IDI participants indicated that CIMCs, particularly heavy or prolonged bleeding, bleeding irregularities, and/or non-bleeding side effects contributed to their desire for removal. Some participants highlighted that heavy bleeding and bleeding irregularities had negatively affected other aspects of their lives, including the ability to be sexually active or participate fully in religious life. A 21-year-old who practices Islam in Kolda explained:

*Before I started using this implant, I used to say my prayers normally but when I started using it, I didn't know how to pray anymore. Because I don't know when I'm going to finish my period and I can resume my prayers … it really messed things up and that's what prompted me to remove it.*

A few participants reported feeling concerned about their health upon experiencing CIMCs or non-bleeding side effects. A combination of side effects and amenorrhea caused a few others to worry about method efficacy and to want to check for pregnancy. Another 29-year-old with 2 children in Dakar Centre shared:

Participant*: Bleeding all this time scared me. I thought the rods were no longer where they had been placed.*

Interviewer*: Did you think about removing it in these moments?*

Participant*: Yes, in fact I did think about removal.*

Several participants wanted to get pregnant. Some participants sought removal because they believed their implant would stop being effective well before its expiration date; another woman lost track of her implant’s expiration date. Several participants, all from Saint Louis, requested a removal due to anticipatory fear of side effects perpetuated through rumors or social media.

Many IDI participants described some degree of partner influence in their removal decision; however, the role and level of partner involvement varied. While some participants explained making the final decision themselves, they reported considering their partner in their decision. Among those reporting direct partner involvement in decision making, most shared that their partner actively encouraged removal, with only a few reporting their partner encouraged keeping the implant to manage family size. Largely, partners recommended removal due to concern for their wife’s health from CIMCs and non-bleeding side effects. For example, a 37-year-old participant with 3 children in Saint-Louis explained:

*He suggested that I remove it because he worried about the possible negative consequences that prolonged bleeding may have. This is the main reason I went to see the midwives for removal services.*

Additional reasons partners encouraged removal included desire for another child, objection to family planning, and frustration with prolonged bleeding interfering with sexual activity.

Several of the participants who had not yet seen a provider despite expressing wanting a removal had nuanced experiences related to needing to find the right time to visit the facility. This included waiting for their next regular appointment, gathering money to pay for services, and finding the appropriate time to leave their house discreetly in the case of a covert user.

#### Access to Removal Services

Most participants cited at least 1 barrier in accessing removal care, often discussing 2 or more. Several participants, especially in Saint Louis, remarked that distance or transport to the facility was a challenge. Additionally, participants frequently explained that their work or domestic obligations constrained their ability to access care; however, all who mentioned this were able to overcome this challenge.

Most participants cited at least 1 barrier in accessing removal care, often discussing 2 or more.

Many participants described a lack of available services upon reaching the health facility, particularly in Dakar Centre. Participants often reported being told that the provider who could perform the service was not present or too busy to see them. Two participants explained that providers were “on strike,” with one waiting 3 months for the strike to end to have her implant removed. Echoing other reports of challenges related to provider availability, a 33-year-old participant from Dakar Centre explained:

*When I came back the second time, she told me that the person who was to perform the removal was not there, she had not come, so she gave me another appointment. When I came back the third time, she told me that the midwife has a lot of sick people. She can't do a removal because it takes time, you have to wait until Monday or Tuesday and come back. This is when I got mad.*

A few participants were unable to have their implant removed due to lack of consumables like anesthetic. Many reported paying for supplies in addition to the service fee.

#### Interactions With Providers

Respondents who sought removal due to rumors about the method or concerns about method expiration were satisfied with the reassurance provided through counseling. One 39-year-old participant from Saint-Louis explained:

*Once in the consultation room, [the midwife] let me know that this was just “fake news” that I shouldn’t believe. And after this exchange, I was reassured, and I left edified.*

Many seeking removal in response to CIMCs or non-bleeding side effects reported being satisfied with counseling or treatment when offered at a first or second visit but described feeling frustrated when the same solution was offered at subsequent visits for persistent issues. A 24-year-old participant from Dakar with “excessive bleeding” made 4 removal attempts whereby she was offered a new prescription at each visit. She conveyed annoyance but said that she was ultimately satisfied to find a treatment that alleviated her CIMC and allowed her to keep her implant. A few participants reported that their provider did not want to remove their implant due to their age or duration on the method.

Regardless of removal outcome, several participants explained that how a provider interacted with them influenced their satisfaction with removal services. A couple described having “confidence” in their provider’s medical assessment, with a 24-year-old in Dakar Centre noting:

*I am absolutely sure that if the midwife had seen danger lurking over me, she would in no way have opposed my attempts for removal.*

Regardless of removal outcome, several participants explained that how a provider interacted with them influenced their satisfaction with removal services.

Another experiencing CIMCs received a treatment that was ineffective but still expressed appreciation that her provider was compassionate about the challenges she faced. Several participants who expressed dissatisfaction with services highlighted negative provider interactions as a contributor, with a 27-year-old from Saint-Louis noting:

*The providers wanted to make a decision for me, something I don’t approve of at all.*

#### Removal Procedures

Among the 11 participants who had a removal, most described the procedure positively. Several noted that they appreciated being shown the implant rods after removal, with a 26-year-old participant in Dakar remarking:

*The most satisfying thing about all this is the communication, that is to say the fact that she showed me the 2 rods after removing them.*

Three participants felt they had a negative removal experience, all of whom had a difficult removal. In these cases, the provider struggled to remove the rod(s) which meant the procedure took longer and caused pain to the participants. A 30-year-old mother of 3 in Saint Louis described the experience:

*You know to remove it, you have to tear it up. She tore it a little, there was blood, and she pulled it out.*

## DISCUSSION

Participant experiences with both implant and IUD removals were primarily positive. Given past research showing that negative experiences with removals can affect method reputation and subsequent uptake,^11^^,^^12^^,^^21^ this bodes well for continued popularity of LARCs. Overall, there were many parallels between the experiences of implant and IUD users, indicating that similar strategies can be deployed to address potential areas of strengthening across LARCs. More IUD users than implant users were satisfied with the outcome of their removal attempt. Among participants who had their method removed, IUD users reported pain or that the provider mentioned difficulties during the procedure less frequently compared to implant users. More IUD users also rated their overall removal-seeking experience as “easy.” This confirms that IUD removals are generally clinically easier than implant removals and also quicker to perform, on average.^22^

The findings highlight some areas for improvement at several points along the participant’s journey to accessing removal services, including improved counseling. Survey results indicate that implant users may not have complete knowledge of their selected method, as evidenced by the incomplete or inconsistent information on method characteristics reported by participants, including the number of rods, duration of protection, or implant name. Ensuring the information given to clients is correct, comprehensive, and easy to understand is at the foundation of informed choice and can be strengthened with well-designed counseling tools. Additionally, CIMCs are an important concern during method use, and together with desired pregnancy, the main reasons for wanting an implant or IUD removal.^23^^–^^27^ Our findings show that CIMCs caused concerns about health and that both CIMCs and other side effects affected participants’ daily lives. In the multivariable analysis, experiencing amenorrhea, experiencing non-bleeding side effects other than weight gain, and being comprehensively counseled on method choice and side effects at insertion were significantly associated with asking for a removal. In contrast, experiencing shorter or reduced bleeding was significantly associated with not asking for a removal, which aligns with other research showing nuanced acceptability of different kinds of CIMCs.^24^ Side effect counseling should be reinforced, inclusive of both anticipatory guidance on CIMCs at insertion and reassurance counseling during follow-up visits.

The findings highlight some areas for improvement at several points along the participant’s journey to accessing removal services, including improved counseling.

Removal decisions mostly took place before the interaction with a provider, though qualitative and quantitative findings differ slightly in the role of partners in decision making. Survey results suggest that many participants decide to seek a removal on their own; however, qualitative data highlight the many ways partners influence removal decisions, including their indirect effect on decision making. This finding supports research from other sub-Saharan countries showing the value of engaging male partners in interventions targeting contraceptive decision making.^28^^–^^31^

Participants also faced challenges before being able to see a provider. On the demand side, this included challenges leaving their home, traveling long distances, or finding money to pay for transport or services. Other difficulties occurred on the service delivery side, with participants encountering long lines or wait times and a lack of available trained providers. These obstacles contributed to participants having to make multiple visits to the health center and delayed removal.

Satisfaction with the outcome of interactions with providers ranged between 63% and 73% at first attempt and exceeded 85% for the most recent interaction by the time of the survey, showing relatively high but not universal levels of satisfaction. Notably, most participants who kept their method after seeing a provider were dissatisfied. Qualitative results highlighted that respondents felt particularly dissatisfied with keeping their implant when their experience of CIMCs or non-bleeding side effects remained unresolved. IDIs also underscored the importance of client-centered care. More research is needed to better understand how providers manage the balance between clients’ autonomy and feelings that early removals may put them at risk of pregnancy, as has been noted elsewhere.^13^^,^^16^^,^^32^ In some cases, participants who had their method removed reported being dissatisfied. Future research should also seek to deepen understanding of these cases to establish whether they indicate disappointment at having to remove the method for a legitimate medical reason, point at areas for improvement in care provision, or uphold reproductive autonomy from partners. Moreover, survey results indicate missed opportunities for reinsertion or method switching at the time of removal. One-third of implant users and one-quarter of IUD users who had their method removed for reasons other than a desired pregnancy left the clinic without another method. Results highlight the importance of the availability of qualified providers in public health facilities and the provision of comprehensive counseling, inclusive of messaging around voluntary discontinuation, method switching, and reinsertion.

When a clinical removal procedure was attempted, the success rate was high. However, some participants, especially implant users, experienced lasting pain following removal. Several scenarios may lead to difficult removals, including weight gain and non-palpable implants or IUDs with non-visible strings.^33^^–^^36^ We found some evidence of difficult implant removals in just under one-fifth of implant users. However, there were only 2 reports of incomplete clinical removal procedures for implant users and no reports for IUD users; furthermore, difficult removals were largely managed successfully. Management of difficult cases will continue to warrant attention as demand for removals increases.

Most participants had not been told about removal costs at the time of insertion. Average removal costs were 34% higher than insertion costs for implants and 17% higher for IUDs. Both insertion and removal costs were also slightly higher for implants. In Senegal, public sector clients are required to purchase a “ticket” covering service fees. The cost of the ticket is not regulated, but it is generally expected not to exceed CFA500 (US$0.85). Our results show that participants paid more and, furthermore, that reported costs varied across participants. Similar findings have been reported elsewhere^14^^,^^16^ and may be explained by the fact that clients can be asked to purchase consumables, like gauze and gloves, when these are not available, and that availability of consumables varies across facilities, as well as within facilities over time. The cost of contraceptive commodities is fixed by the Ministry of Health and is not supposed to be transferred to clients; however, this guidance is not always applied. To address the burden of the cost of removal, guidance should be put in place to harmonize service fees and other costs across all public health facilities. Procurement and funding mechanisms for supplies also warrant attention to ensure that costs are not unduly passed on to clients and that removal services remain affordable. Additionally, counseling tools can be adapted to include information about removal costs at time of insertion.

### Limitations

The purposive selection of districts and the need for phone information carries some risk of selection bias. The study population may not be representative of the general LARC user population and may exclude those hiding use from their partner and the poorest women who are likely to experience greater barriers to removal including financial barriers. We used a 2-step design to exclude women who had never asked a provider for a removal and allow time for recall of their experiences between the 2 steps for those who did. However, this process also resulted in attrition between the 2 interview rounds. Additionally, attrition may have been compounded by the fact that the interval between the 2 surveys was longer than initially intended due to practical reasons related to the organization of fieldwork. Sample size for the regression model is reduced due to missing data on covariates. IDI participant selection prioritized allowing understanding of barriers to removals and was not designed to elucidate why some participants who had their implant removed reported not being satisfied with this outcome.

## CONCLUSION

Findings showing largely satisfactory removal outcomes are encouraging and point to mostly similar experiences across implants and IUDs. These results are important to fulfill the aims of voluntary family planning and informed choice, inclusive of method discontinuation, in support of a rights-based approach to care and for the continued success of LARCs in sub-Saharan Africa. Areas of potential improvement to further strengthen access to removal services in Senegal include client flow, counseling messages at insertion and when advising clients to keep their method, and pricing. Additionally, ensuring access to methods for reinsertion or method switching after removal could increase contraceptive continuation.

## Translation

**En Français**

**PRENDRE EN COMPTE LES RETRAITS DANS LE CHOIX ÉCLAIRÉ : UNE ÉTUDE À MÉTHODES MIXTES SUR LES EXPÉRIENCES DES CLIENTES AVEC LE RETRAIT DES CONTRACEPTIFS RÉVERSIBLES À LONGUE DURÉE D'ACTION AU SÉNÉGAL**

**ABSTRAIT**

**Contexte:** Garantir l'accès aux services de retrait d'implants et de dispositifs intra-utérins (DIU) est essentiel pour permettre le choix éclairé et la planification familiale volontaire. Nous examinons ici les désirs et les expériences de retrait chez les femmes qui ont reçu un implant ou un DIU du secteur public dans 3 districts du Sénégal.

**Méthodes:** Nous avons mené une enquête téléphonique auprès de 1 868 utilisatrices d'implants et de DIU, 598 enquêtes de suivi auprès de celles qui avaient déjà demandé un retrait à un prestataire et 24 entretiens approfondis (EA) avec des femmes qui avaient déjà souhaité un retrait d'implant. Nous avons analysé les données d'enquête de manière descriptive et les données des EA de manière thématique.

**Résultats:** Cinquante-huit pourcent des utilisatrices d'implants et 54% des utilisatrices de DIU ont déclaré avoir souhaité un retrait. Le désir de grossesse et les changements menstruels induits par la contraception (CMIC) étaient les principales raisons pour désirer un retrait. Cinquante-quatre pourcent des utilisatrices d'implants et 55% des utilisatrices de DIU qui ont demandé un retrait à un prestataire ont signalé des difficultés d'accès aux services, plus des deux tiers faisant état de longues queues ou de longues durées d'attente. Soixante-trois pourcent des utilisatrices d'implants et 73% des utilisatrices de DIU qui ont consulté un prestataire étaient satisfaites du résultat de leur première interaction avec celui-ci. Plus de 90% des participantes n'avaient pas été informés du coût du retrait lors de l'insertion de leur méthode. Presque toutes les participantes dont la méthode a été retirée ont obtenu un retrait complet lors de leur première intervention clinique. Environ les deux tiers des participantes qui ont obtenu un retrait n'ont pas adopté d'autre méthode à ce moment-là. Les EA ont confirmé l'influence des CMIC sur les désirs de retrait et montrent qu'une certaine influence du partenaire est courante dans la prise de décision liée au retrait. Les obstacles incluent le manque de prestataires qualifiés et la disponibilité limitée des fournitures. Les interactions avec les prestataires jouent un rôle important dans la satisfaction à l'égard des services de retrait.

**Conclusion:** Les expériences des participantes en matière d'accès aux services de retrait étaient généralement positives. Les domaines d'amélioration potentiels incluent le flux de clientes, le counseling lors de l'insertion et lorsqu'il s'agit de conseiller aux clientes de conserver leur méthode, les prix, et la réinsertion après le retrait ou le changement de méthode.

**Peer Reviewed**

**First published online:** September 19, 2022.
